# CTCF regulates the FoxO signaling pathway to affect the progression of prostate cancer

**DOI:** 10.1111/jcmm.14138

**Published:** 2019-03-15

**Authors:** Zhengfei Shan, Yongwei Li, Shengqiang Yu, Jitao Wu, Chengjun Zhang, Yue Ma, Guimin Zhuang, Jiantao Wang, Zhenli Gao, Dongfu Liu

**Affiliations:** ^1^ Department of Organ Transplantation The Affiliated Yantai Yuhuangding Hospital of Qingdao University Yantai Shandong China; ^2^ Department of Urology The Affiliated Yantai Yuhuangding Hospital of Qingdao University Yantai Shandong China

**Keywords:** CTCF, FoxO signaling pathway, prostate cancer

## Abstract

The present research focuses on the influence of CCCTC‐binding factor (CTCF) on prostate cancer (PC) via the regulation of the FoxO signalling pathway. A bioinformatics analysis was conducted to screen out target genes for CTCF in LNCaP cells and to enrich the relevant pathways in LNCaP cells. It was found that the FoxO pathway was enriched according to the ChIP‐seq results of CTCF. The expression of CTCF, pFoxO1a, FoxO1a, pFoxO3a and FoxO3a was tested by RT‐qPCR and Western blot. Inhibition of CTCF could lead to the up‐regulation of the FoxO signalling pathway. The rates of cell proliferation, cell invasion and apoptosis were examined by MTT assay, cell invasion assay and flow cytometry under different interference conditions. Down‐regulation of CTCF could suppress cell proliferation, cell invasion and facilitate cell apoptosis. Lastly, the effect of CTCF on tumour growth was determined in nude mice. Inhibition of CTCF regulated the FoxO signalling pathway, which retarded tumour growth in vivo. In conclusion, CTCF regulates the FoxO signalling pathway to affect the progress of PC.

## 1 INTRODUCTION

Among men in the United States, prostate cancer (PC) is one of the most common diseases and the second leading cause of cancer‐related deaths. The pathogenesis of PC is still unknown to us. However, several risk factors such as ethnicity, family history and age are related to the disease.^1^, ^2^ Furthermore, some dietary components have been found to be related to the risk and prevention of PC.^3^, ^4^ When PC reaches an advanced stage, clinical treatments such as surgery, androgen deprivation and radiotherapy may exert little effect on androgen‐independent PC, which is associated with a 2‐3 year life expectancy. Although the morbidity and mortality of PC has received much attention in recent years, metastasized PC remains incurable and effective therapies are urgently needed.^5^ The progression of PC is related to epigenetic changes in both normal and cancerous tissues.^6^ The factors affecting these changes are still unknown. One of the relevant factors associated with the regulation of epigenetic marks of PC is the CCCTC‐binding factor (CTCF).^7^

The CCCTC‐binding factor (CTCF) is an evolutionarily conserved 11‐zinc finger protein that acts as a fundamental factor in physiological regulatory activities, including transcriptional activation/repression, insulating, imprinting as well as X chromosome inactivation.^8^ There are more than 20 000 binding sites in the CTCF genome; therefore, the regulatory actions of CTCF are quite complex and depend on the specific DNA sequence and interacting factors at CTCF binding sites.^9^ The distribution of CTCF binding sites in the genome relates to gene density, with approximately 46% of sites lying in intergenic regions, 20% near transcriptional start sites, 22% in introns and 12% in exons.^10^ CTCF is a nuclear protein, which is widespread across cell types. Dysfunction of CTCF can epigenetically alter many cancer‐related genes. Recent genome‐wide assays have demonstrated that the transcription factor CTCF can link chromatin domains through long‐distance interactions between distal genomic regions, suggesting a critical role in chromatin conformation.^11^

FoxO proteins, including FoxO1a and FoxO3a, are evolutionarily conserved transcription factors that are involved in multiple fundamental cellular activities, acting in transcriptional activities related to cell proliferation, apoptosis and stress response.^12^, ^13^, ^14^, ^15^, ^16^ Numerous therapies can induce cell growth arrest and apoptosis through activation of FoxO transcription factors in PC cells.^17^ However, upexpression of FoxO has inhibited tumorigenesis in xenograft models in nude mice.^18^, ^19^, ^20^, ^21^, ^22^, ^23^, ^24^ Therefore, reactivation of FoxO based on its tumour‐suppressant properties is considered a very promising therapy for PC. Since FoxO proteins have been found to be critical mediators of apoptosis, we hypothesized that FoxO expression or its transcriptional activity could be an important event in changing the progression of PC.

Therefore, we studied the relationship between CTCF and FoxO signalling. To assess the rates of cell proliferation, cell invasion and apoptosis, an MTT assay, cell invasion assay and flow cytometry were performed under different interference conditions. The flow cytometry detected the effect of CTCF on tumour growth in nude mice.

## 2 MATERIALS AND METHODS

### 2.1 Bioinformatics analysis

A microarray including ChIP‐seq of normal and cancerous prostate cells (PrEC, LNCaP) was downloaded from GEO (Gene Expression Omnibus, https://www.ncbi.nlm.nih.gov/geo/; GSE38684). DAVID was used for the GO and KEGG pathway enrichment analyses (https://david.ncifcrf.gov).

### 2.2 Cell culture and tissue sample collection

Normal human prostate epithelial cells, PrECs, were obtained from Clonetics Corporation, San Diego, CA, USA. PrECs were grown in a serum‐free PrEGM medium with supplements provided by Clonetics Corp. The established human PC cell lines of LNCaP and PC‐3 were obtained from the American Type Culture Collection, Rockville, MD, USA.

With RPMI 1640 medium and 10% FBS (Gibco BRL, Life Technologies), all cancer cell lines were cultured at 37°C in an atmosphere of 95% air and 5% CO_2_. The cell lines were subcultured three times a week, and the medium was replaced every 2 days. No antibiotics were used during culturing of the cells.

Fifteen primary human tumour tissues, together with adjacent tissues, were collected during surgery from breast cancer patients treated at Colchester General Hospital, with written consent obtained prior to surgery. The research was approved by the ethics committee of The Affiliated Yantai Yuhuangding Hospital of Qingdao University. Tumour tissues were selected by conventional pathologic criteria, and the histopathology was further confirmed by microscopic examination.

### 2.3 Transfection

Scramble siRNA was used as a control (GenePharma, Shanghai, China). CTCF siRNA was obtained from GenePharma, Shanghai, China. The transfection procedure was performed using Lipofectamine RNAiMAX (Life Technologies) following the manufacturer's protocols.^25^

### 2.4 RT‐qPCR

By using TRIzol reagent (Invitrogen Life Technologies, Carlsbad, CA, USA), total RNA was extracted 48 hours after transfection. RT‐qPCR was performed using MonsterScript^™^ Reverse Transcriptase (Epicentre, Illumina, Inc., Madison, WI, USA) and SYBR^®^ Green I nucleic acid gel stain (Molecular Probes Life Technologies, Carlsbad, CA, USA). The results were determined using the 2^−ΔΔCt^ method. GAPDH was used as a control for normalization, and the data were calculated and analysed with Rotor‐Gene Real‐Time Analysis software 6.0 (Corbett Research, Mortlake, Australia).

### 2.5 Western blot

Samples containing 20 μg of total protein were separated on 8%‐12% SDS‐PAGE gels according to the different molecular weights and then transferred onto nitrocellulose membranes (Whatman, Germany) in transfer buffer using a Mini Trans‐Blot Cell (Bio‐Rad) at 400 mA for 2 hours. The membranes were blocked by incubating them in 5% nonfat milk in TBS‐T for 1 hour at room temperature. Proteins were detected using specific rabbit polyclonal anti‐CTCF (1:1000, ab203312; Abcam, USA), rabbit polyclonal anti‐pFoxO1a (1:200, ab131339; Abcam), rabbit polyclonal anti‐FoxO1a (1:500, ab70382; Abcam), rabbit polyclonal anti‐pFoxO3a (1 μg/mL, ab47285; Abcam), rabbit polyclonal anti‐FoxO3a (5 μg/mL, ab23683; Abcam), rabbit polyclonal anti‐Lamin B1 (1 μg/mL, ab65986; Abcam) and rabbit polyclonal anti‐GAPDH (5 μg/mL, ab9485; Abcam) antibodies. After being washed with TBS‐T, the membranes were incubated with goat anti‐rabbit immunoglobulin G secondary antibodies (1:50 000, ab205718; Abcam) in TBS‐T containing 5% nonfat milk for 45 minutes at room temperature. The grey value of bands was analysed by enhanced ImageJ (Version 1.48u; MD, USA).

### 2.6 MTT assay

Cell viability was measured using microculture tetrazolium test (MTT) from Sigma‐Aldrich as described in the manufacturer's manual. Briefly, the cells that had been transfected for 0, 12, 24 and 48 hours were added to 20 μL of 5 mg/mL MTT and then incubated at 37°C for 4 hours. The medium was removed, and 150 μL of the MTT solution was added to the cells for a further incubation of 15 minutes at room temperature with shaking.

### 2.7 Cell invasion assay

Cell invasion was assessed using the Transwell assay. LNCaP and PC‐3 cells were harvested after trypsinization and washed with a serum‐free media containing 0.1% bovine serum albumin. Cells were diluted to 5 × 10^5^ cells/mL, and 100 μL of the cell suspension was seeded on the top of Matrigel invasion chambers (8 μm pore size, cat no. 354480; Corning). The lower chamber was filled with 600 μL of medium containing 10% FBS. After being incubated for 24 hours at 37°C, cells from the upper chamber were gently removed with a cotton swab. The filter was fixed with 4% paraformaldehyde for 15 minutes at room temperature, then stained with 500 μL of 0.1% crystal violet for 15 minutes, and washed with PBS, and the cells were counted under a light microscope to determine the level of cell invasion. Each cell type was assayed in triplicate, and all the experiments were repeated three times.

### 2.8 Wound healing assay

LNCaP and PC‐3 cells transfected with siNC, siCTCF were cultured to obtain 80%‐90% monolayer confluency. A wound was created by scraping the cells using a 10 μL plastic pipette tip, and the old medium was replaced with fresh medium. Cells were photographed under a microscope equipped with a camera at different points in time.

### 2.9 Flow cytometry

The number of apoptotic cells was calculated using an Alexa Fluor^®^ 488 annexin V/Dead Cell Apoptosis Kit with Alexa^®^ Fluor 488 annexin V and PI for flow cytometry (Invitrogen) as described in the manufacturer's manual. The protocol was carried out according to a previously described method.^26^ Cells were analysed by fluorescence‐activated cell sorting using Becton Dickinson FACSCalibur cell sorter and CellQuest software. The results show that the percentage of apoptotic cells was relevant to the total cell number.

### 2.10 Tumour formation in nude mice

Nude mice (4‐5 weeks old, 14‐16 g) were purchased and housed in barrier facilities with a 12 hours/12 hours light/dark cycle.^27^ All experiments with nude mice were executed under institutional guidelines and approved by the Institutional Animal Care and Use Committee. Mice were divided randomly into four groups (n = 4/group). For tumour cell implantation, 5 × 10^6^ LNCaP or PC‐3 cells (100 μL volume) transfected with siNC and siCTCF were injected subcutaneously into the mice. Tumours were detected on day 7 after injection and then examined once every week. Tumour length, width and thickness were measured using a caliper, and tumour volume was calculated by the ellipsoid volume calculation formula: 0.5 × (length × width^2^). At day 28 after injection, animals were killed, and the tumours were excised and weighed.

## 3 RESULTS

### 3.1 Bioinformatics analysis

**Figure 1:**
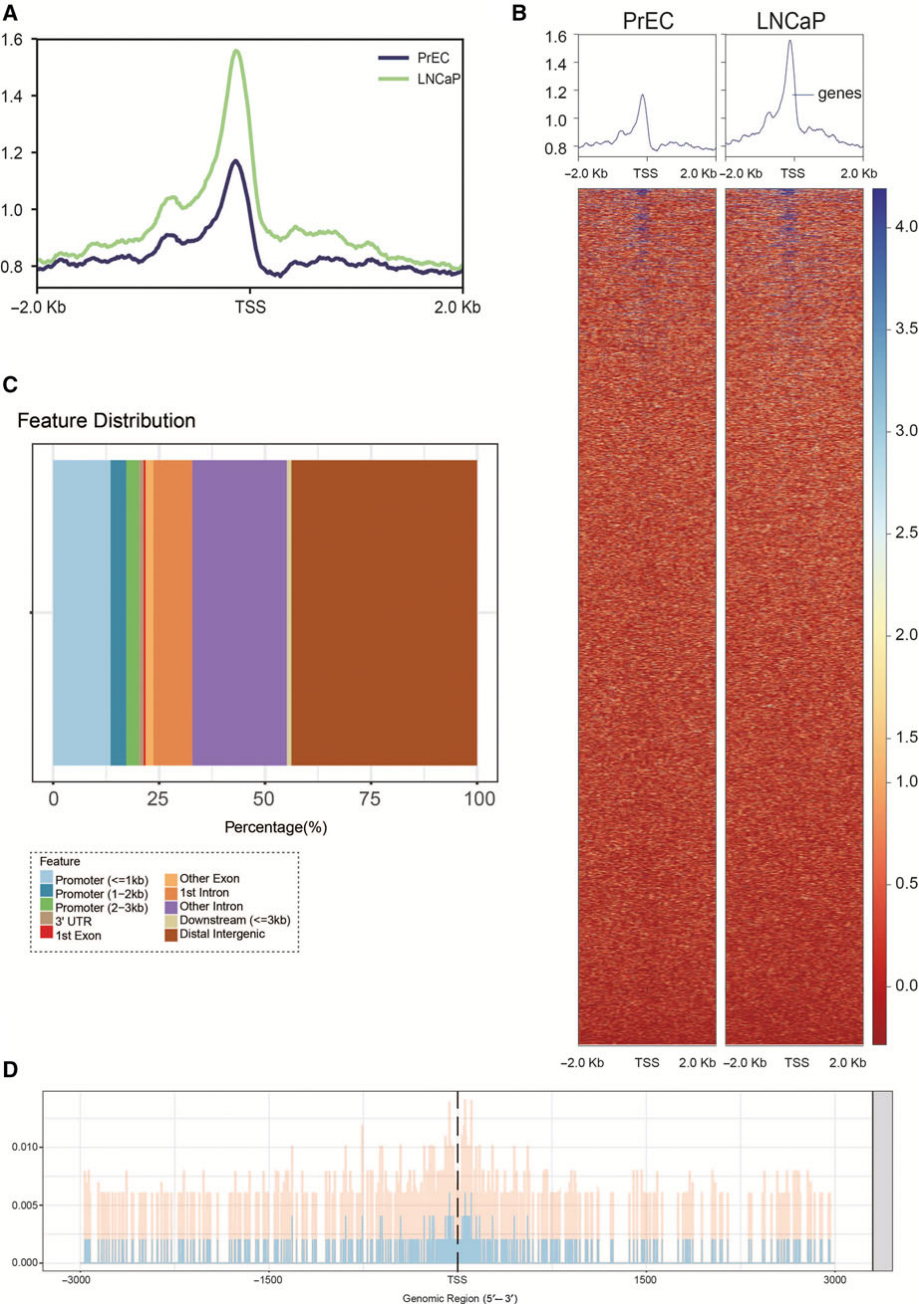
ChIP‐seq analysis. A and B, Gene binding site of transcription factor CTCF was enriched near TSS region in PrEC and LNCaP cells. C, Feature distribution of CTCF genome. D. Details of CTCF binding to TSS region

Analysis of ChIP‐seq data showed the gene binding site of transcription factor CTCF was enriched near the TSS region in PrEC and LNCaP cells. The enriched peak in LNCaP cells was higher than that in PrEC cells, which revealed that CTCF and CTCF‐related genes might have a strong connection with PC (Figure 1A,B). The feature information of the CTCF genome is shown in Figure 1C. Details of CTCF binding to the TSS region are shown in Figure 1D.

### 3.2 Gene functional analysis by ChIP‐seq

**Figure 2:**
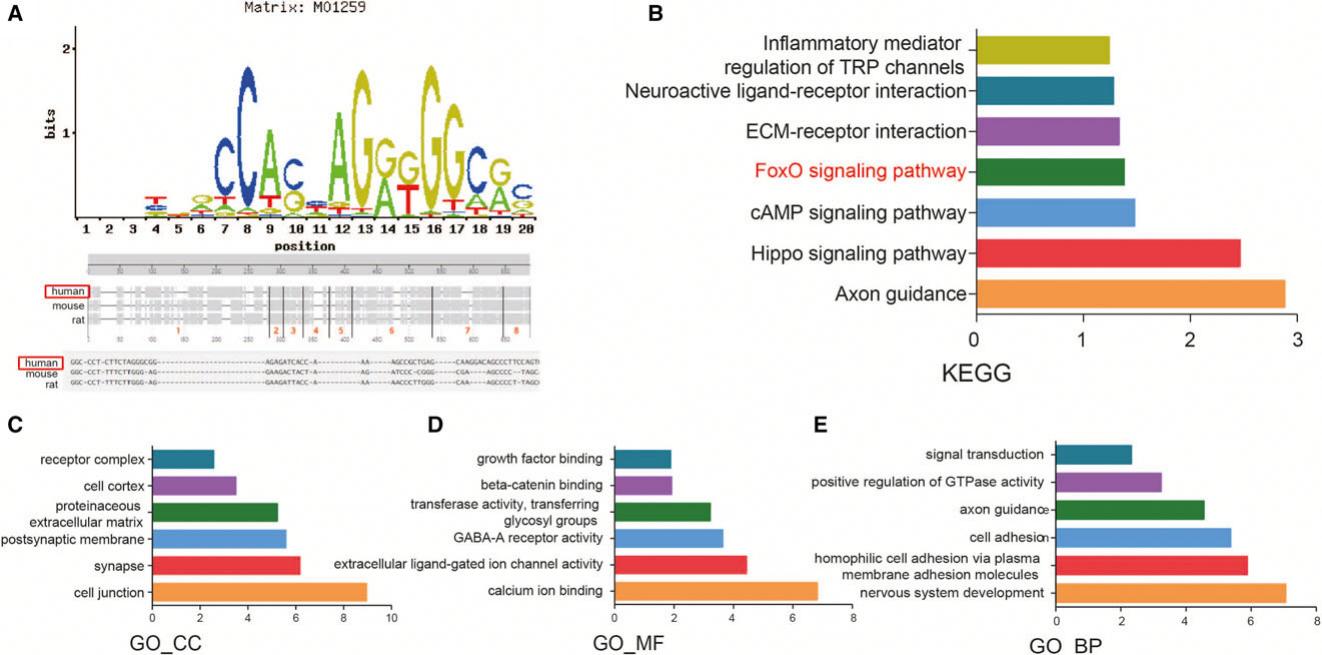
Gene functional analysis by ChIP‐seq. A, Motif of CTCF predicted on website. Possible binding region was compared among humans, mice and rats. B, KEGG analysis of genes from ChIP‐seq analysis was performed on DAVID. C‐E, GO analysis of genes from ChIP‐seq analysis was performed on DAVID

To determine the possible binding region of CTCF in different species such as humans, mice and rats, the motif was predicted on a website (Figure 2A). KEGG analysis of genes from ChIP‐seq analysis was performed on DAVID (Figure 2B). GO analysis of genes from ChIP‐seq analysis was also performed on DAVID. According to the analyses, CTCF was related to biological processes, such as signal transduction, and was located in synapses, cell junctions and many other areas. Furthermore, it could exert multiple functions such as growth factor binding and GABA‐A receptor activity (Figure 2C‐E). Multiple signalling pathways were involved, and investigating the FoxO signalling pathway was regarded as our research objective.

### 3.3 CTCF was dysregulated in LNCaP

**Figure 3:**
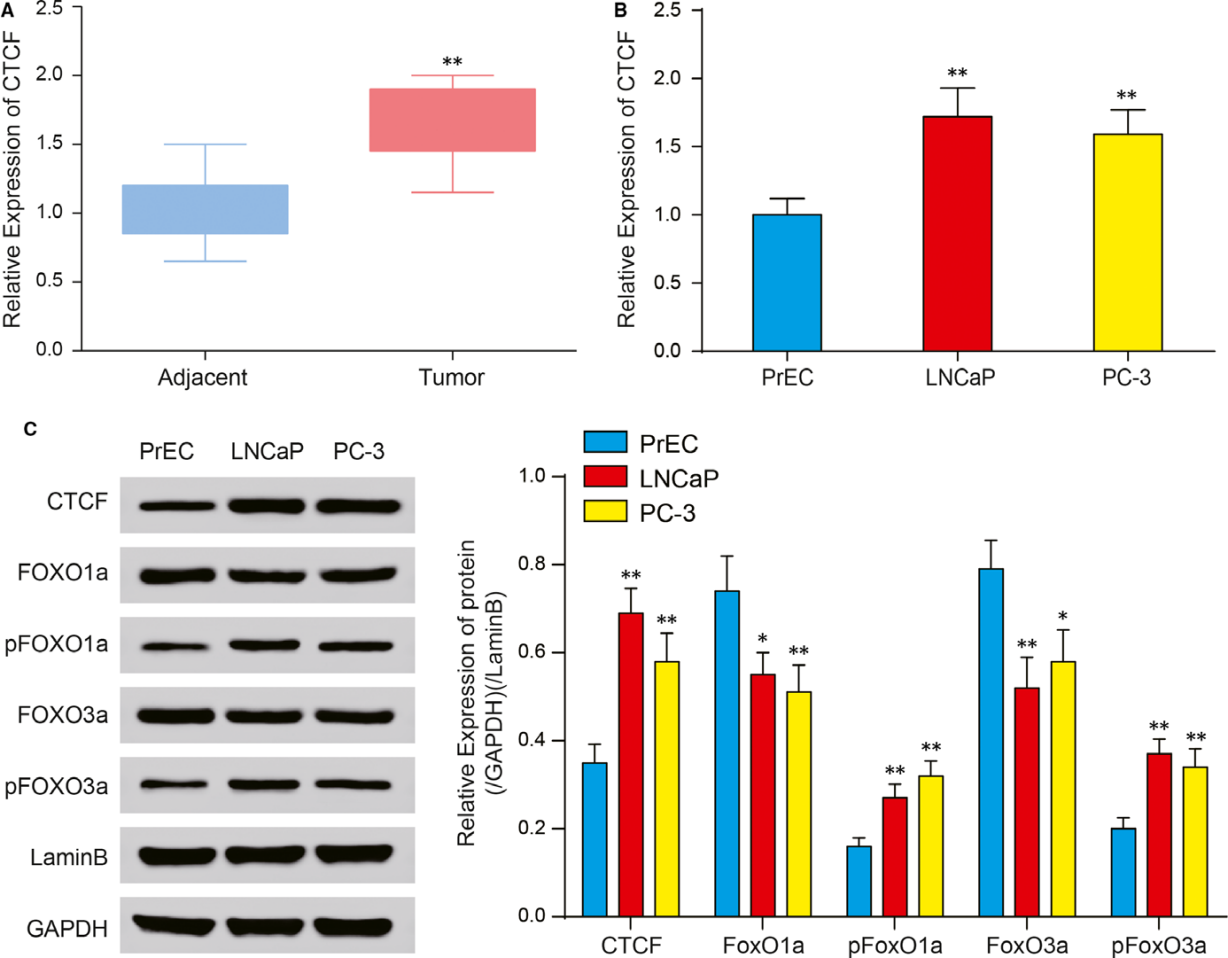
Expression of CTCF. A, RT‐qPCR showed that the expression of CTCF mRNA was up‐regulated in tumour tissues compared to tissues adjacent to tumours. B, RT‐qPCR showed that the expression of CTCF mRNA was up‐regulated in LNCaP and PC‐3 cells compared to PrEC. C, Western blot indicated that the expression of CTCF, pFOXO1a and pFOXO3a was high in LNCaP and PC‐3 cells, while that of FOXO1a and FOXO3a was low. PrEC is a prostate epithelial cell line; LNCaP and PC‐3 are prostate cancer cell lines. *P < 0.05, **P < 0.01, compared with PrEC group

To determine the expression of CTCF in tumours and adjacent tissues, PrEC, LNCaP and PC‐3 cells, qPCR and Western blot tests were conducted. The results showed that compared with tissues in cells adjacent to the tumours, the mRNA of CTCF was richly expressed in tumour tissues (Figure 3A). The expression of CTCF was up‐regulated in LNCaP and PC‐3 cells compared with PrEC (Figure 3B). Western blot tests showed that the expression of CTCF, pFOXO1a and pFOXO3a was up‐regulated, while that of FOXO1a and FOXO3a was down‐regulated (Figure 3C) in LNCaP and PC‐3 cells.

### 3.4 CTCF promoted the proliferation of PC cells by regulating the FoxO signalling pathway

**Figure 4:**
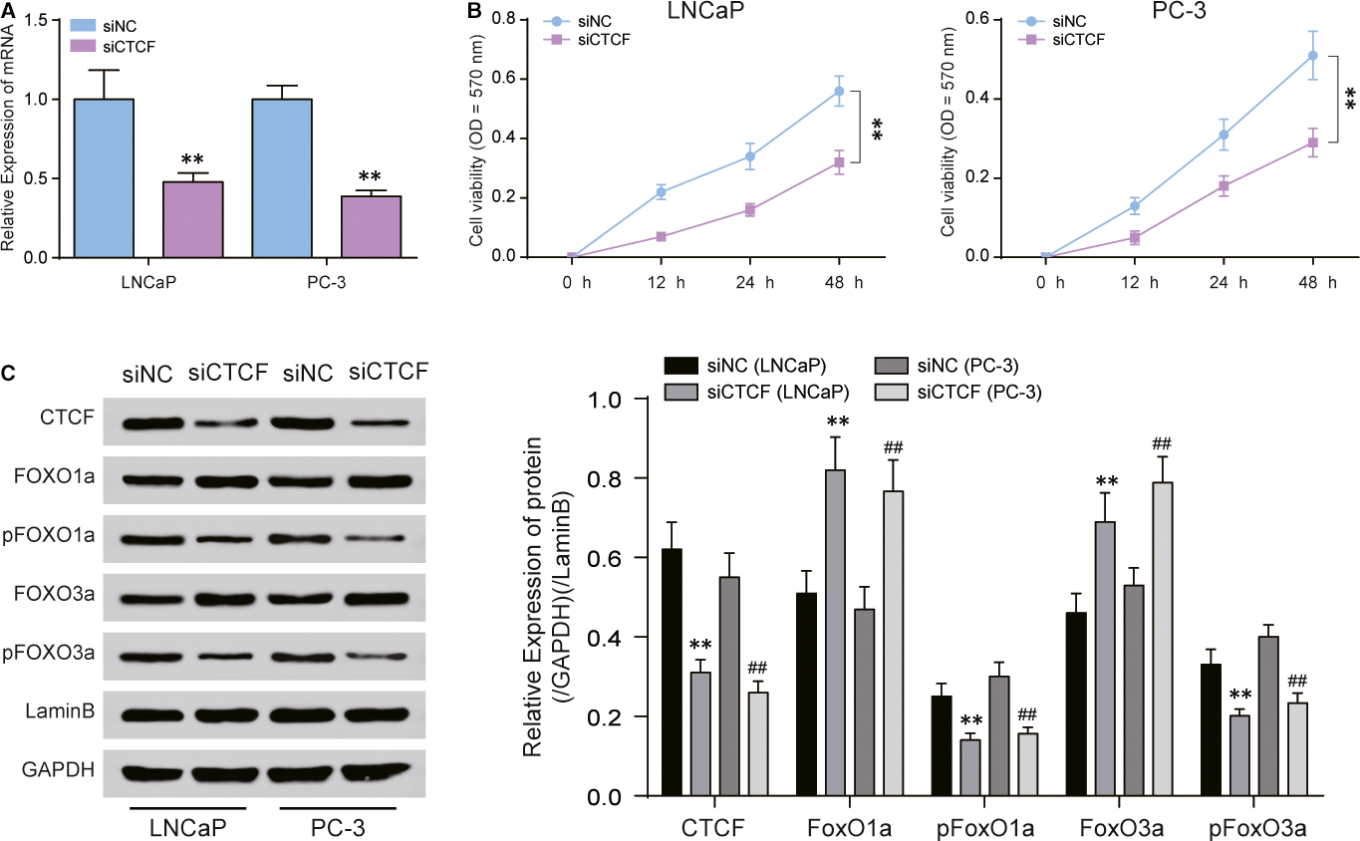
CTCF regulates FoxO signalling pathway to affect the proliferation of prostate cancer. A, RT‐qPCR revealed the mRNA expression of CTCF in LNCaP and PC‐3 cells by siCTCF. B, MTT assay showed that the cell proliferation of LNCaP and PC‐3 cells under the treatment of siCTCF. C, Western blot showed the expression of CTCF, FOXO1a, pFOXO1a, FOXO3a and pFOXO3a in LNCaP and PC‐3 cells. **P < 0.01, compared with siNC group. ##*P* < 0.01, compared with siNC group

**Figure 5:**
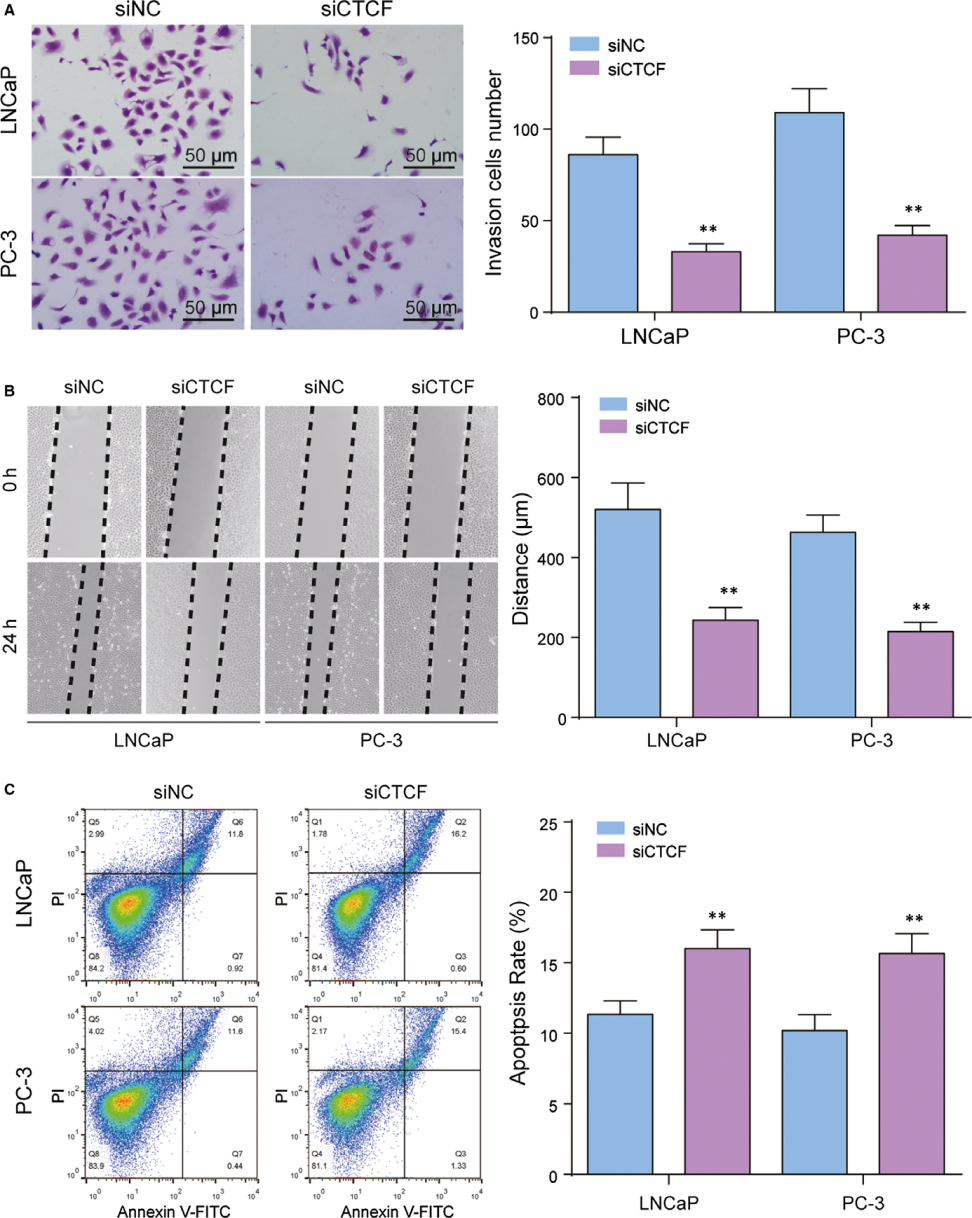
CTCF regulates the FoxO signalling pathway to affect the invasion and apoptosis of prostate cancer cells. A, Cell invasion was examined by transwell experiment. B, Cell metastasis was tested in LNCaP cells by wound healing experiment. C, Cell apoptosis of LNCaP and PC‐3 was examined by flow cytometry. **P < 0.01, compared with siNC group

CTCF regulates the FoxO signalling pathway to affect the progression of PC. RT‐qPCR revealed that siCTCF inhibited the expression of CTCF distinctly (Figure 4A). MTT assays showed that the inhibition of CTCF could retard the proliferation of LNCaP and PC‐3 cells (Figure 4B). Western blot tests showed that the protein expression of CTCF was inhibited after siCTCF treatment. FoxO1a and FoxO3a are tumour suppressing genes, which deactivate after phosphorylation. After the treatment of siCTCF, the expression of FOXO1a and FOXO3a was up‐regulated, while that of pFOXO1a and pFOXO3a was down‐regulated (Figure 4C). After the application of siCTCF, cell invasion was significantly reduced (Figure 5A), tumour metastasis was suppressed (Figure 5B) and cell apoptosis was promoted in LNCaP and PC‐3 cells (Figure 5C).

### 3.5 siCTCF retarded tumour growth in vivo

**Figure 6:**
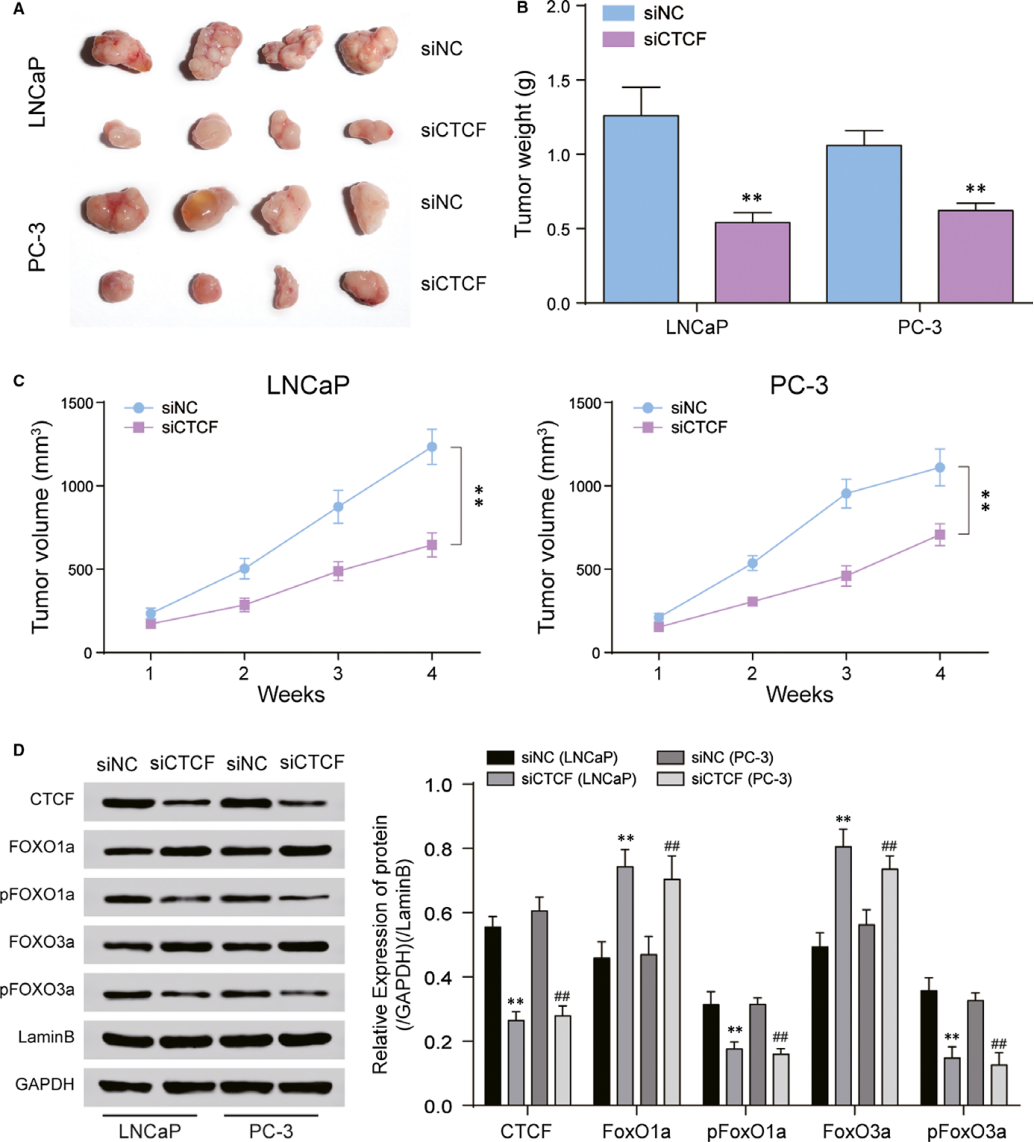
Tumour growth in vivo. A, Tumour tissues in nude mice were collected. B, Tumour weight was reduced by siCTCF. C, Line chart of tumour volume D. Western blot revealed the expression of CTCF, pFOXO1a, FOXO1a, pFOXO3a and FOXO3a in tumour tissues. **P < 0.01, compared with siNC group. ##*P* < 0.01, compared with siNC group

siNC and siCTCF were injected into nude mice, and the tumour tissues are shown in Figure 6A. As seen in Figure 6B, tumour volume in the siNC group had been increasing rapidly, while the growth in tumour volume in the siCTCF group slowed down starting at week 3. At week 4, the tumour volume in the siCTCF group was quite smaller than in the siNC group. Tumour weight in the siCTCF group was quite lighter in the siNC group (Figure 6C). After siCTCF treatment, Western blot revealed that the expression of CTCF, pFOXO1a and pFOXO3a was down‐regulated, while that of FOXO1a and FOXO3a was up‐regulated in tumour issues (Figure 6D).

## 4 DISCUSSION

In this study, analysis of ChIP‐seq data showed that the gene binding site of transcription factor CTCF was enriched near the TSS region in PrEC and LNCaP cells. KEGG pathway enrichment of ChIP‐seq results had enriched several pathways including the FoxO signalling pathway. The FoxO signalling pathway was chosen for further experiments because it was found to be related to PC in previous studies.^28^, ^29^, ^30^ Compared with PrEC, the expression of CTCF was up‐regulated in cancer cells. CTCF regulates the FoxO signalling pathway, affecting the progression of PC. As seen from the MTT assay, cell invasion assay and wound healing assay, inhibition of CTCF could lead to the inhibition of cell proliferation, cell invasion and promotion of cell apoptosis in LNCaP and PC‐3 cells by up‐regulating FoxO1a and FoxO3a in the FoxO signalling pathway. Furthermore, inhibition of CTCF could promote the FoxO signalling pathway and retard tumour growth in vivo. Our research proved that CTCF could retard PC progression by affecting the FoxO signalling pathway.

Prostate cancer is the second leading cause of cancer‐related death among men in the United States.^2^ The factors involved in the progression of PC are not yet fully understood. The correlative factors associated with the regulation of epigenetic marks of PC include the CCCTC‐binding factor (CTCF).^7^ CTCF is an evolutionarily conserved nuclear protein which is fundamental in regulatory activities in numerous cell types.^8^ There are more than 20 000 binding sites in the CTCF genome; therefore, the regulatory actions of CTCF are quite complex and depend on the specific DNA sequence and interacting factors at CTCF binding sites.^9^ In our previous study, analysis of ChIP‐seq data showed that the gene binding site of transcription factor CTCF was enriched near the TSS region in both PrEC and LNCaP cells. The enriched peak in LNCaP cells was higher than in PrEC, which suggested that CTCF and CTCF‐related genes might have a strong connection to PC. This result indicated that compared with tissues adjacent to the tumour, the mRNA of CTCF was highly expressed in tumour tissues. The expression of CTCF was up‐regulated in LNCaP cells compared with PrEC.

Research found that every zinc finger mutation enabled CTCF binding to a subset of target sites within the promoters/insulators of certain genes, which is involved in the reduction of cell proliferation. Therefore, CTCF may represent a new type of tumour suppressor gene. Mutations of CTCF display a selectivity in change of function.^31^ In our study, MTT assays showed that the inhibition of CTCF could retard the proliferation of LNCaP and PC‐3 cells. After being treated with siCTCF, cell invasion decreased significantly, tumour metastasis was inhibited and cell apoptosis was promoted in LNCaP and PC‐3 cells.

KEGG analysis of genes from ChIP‐seq analysis found that CTCF was involved in the FoxO signalling pathway. Studies on PC patients also found the increased cytoplasmic expression of phosphorylated FoxO3a (Ser253), which relates to disease progression.^32^ In addition, a decrease in FoxO function is frequently observed with several human cancers.^15^, ^33^ Data indicate that chemical compounds such as resveratrol could induce apoptosis and growth arrest through activation of FoxO transcription factors.^17^ Moreover, the forced expression of FoxO has been confirmed to inhibit tumorigenesis in xenograft models in nude mice.^19^, ^20^, ^21^, ^22^, ^23^, ^24^ In our present study, after siCTCF treatment, the expression of pFOXO1a and pFOXO3a was up‐regulated, while that of FOXO1a and FOXO3a was down‐regulated in LNCaP cells. After siCTCF treatment, Western blot showed that the protein expression of CTCF in tumour tissues in nude mice was inhibited, the expression of FOXO1a and FOXO3a was up‐regulated, while that of pFOXO1a and pFOXO3a was down‐regulated. Tumour growth was evidently inhibited in nude mice with PC. The therapy approach of targeting CTCF seems to be promising for the treatment of PC by regulating the FoxO signalling pathway and further retarding cancer progression. Additionally, there have been an increasing number of studies on the use of combination therapies to treat cancer using DNA repair mechanisms.^34^, ^35^, ^36^ Research has shown that the inactivation of AKT results in dephosphorylation and activation of FOXO transcription factors, which is involved in mediating cell cycle arrest, DNA repair and apoptosis.^31^, ^32^ It would be feasible to make a combination therapy via DNA repair progression.

In our study, we identified an interaction between CTCF and the FoxO signalling pathway in PC progression. However, some questions remain. The specific mutations of the gene binding regions of CTCF and the importance of the role of CTCF and the FoxO signalling pathway in PC cells warrant further study.

In summary, our research proved that CTCF could retard PC progression by altering the FoxO signalling pathway in vitro and in vivo. These data indicate that CTCF may serve as a potential therapeutic target for PC.

## CONFLICT OF INTEREST

Authors declare no conflicts of interest for this article.

## AUTHOR CONTRIBUTION

Zhengfei Shan, Yongwei Li, Shengqiang Yu, Jitao Wu conceived and designed of the work and acquisition, analysed the data and drafted the article; Chengjun Zhang, Yue Ma, Guimin Zhuang analysed the data and revised the article critically for important intellectual content; Jiantao Wang, Zhenli Gao, Dongfu Liu performed the experiments and analysed the data. All authors read and approved the version to be published, and participated sufficiently in the work to take public responsibility for appropriate portions of the content.
